# Identifying Social Determinants and Measuring Socioeconomic Inequalities in the Use of Four Different Mental Health Services by Australian Adolescents Aged 13–17 Years: Results from a Nationwide Study

**DOI:** 10.3390/healthcare11182537

**Published:** 2023-09-14

**Authors:** Md Irteja Islam, Shumona Sharmin Salam, Enamul Kabir, Rasheda Khanam

**Affiliations:** 1 Sydney School of Public Health, Faculty of Medicine and Health, The University of Sydney, Camperdown, NSW 2006, Australia; 2 Centre for Health Research and School of Business, Faculty of Business, Education, Law and Arts, University of Southern Queensland, Toowoomba, QLD 4350, Australia; rasheda.khanam@usq.edu.au; 3 School of Mathematics, Physics and Computing, Faculty of Health, Engineering and Sciences, University of Southern Queensland, Toowoomba, QLD 4350, Australia; enamul.kabir@usq.edu.au; 4 Bristol Medical School (PHS), The University of Bristol, Bristol BS8 1QU, UK; shumona.salam@bristol.ac.uk

**Keywords:** mental health services, social determinants, socioeconomic inequality, concentration index, adolescents, Australia

## Abstract

Aim: In this study, we aimed to identify the determinants of four different forms of mental health service usage (general health services, school counselling, telephone, and online services), and the number of mental health services accessed (single and multiple) by Australian adolescents aged 13–17 years. We also measured socioeconomic inequality in mental health services’ usage following the concentration index approach within the same sample. Subject and Methods: The data came from the nationwide cross-sectional survey, Young Minds Matter (YMM): the second Australian Child and Adolescent Survey of Mental Health and Wellbeing. Random effect models were used to identify the factors associated with four different mental health services and the number of services accessed. Further, the Erreygers’ corrected concentration indices for binary variables were used to quantify the socioeconomic inequality in each mental health service. The four services were the general health service (GP, specialist, psychiatrist, psychologist, hospital including emergency), school services, telephone counselling and online services. Results: Overall, 31.9% of the total analytical sample (*n* = 2268) aged 13–17 years old visited at least one service, with 21.9% accessing a single service and 10% accessing multiple services. The highest percentage of adolescents used online services (20.1%), followed by general mental health services (18.3%), while school services (2.4%) were the least used service. Age, gender, family type and family cohesion statistically significantly increased the use of general health and multiple mental health service usage (*p* < 0.05). Area of residence was also found to be a significant factor for online service use. The concentration indices (CIs) were −0.073 (*p* < 0.001) and −0.032 (*p* < 0.001) for health and telephone services, respectively, which implies pro-rich socio-economic inequality. Conclusion: Adolescents from low-income families frequently used general mental health services and telephone services compared to those who belonged to high-income families. The study concluded that if we want to increase adolescents’ usage of mental health services, we need to tailor our approaches to their socioeconomic backgrounds. In addition, from a policy standpoint, a multi-sectoral strategy is needed to address the factors related to mental health services to reduce inequity in service utilisation.

## 1. Introduction

Adolescent mental health is a global concern with evidence indicating an inverse relationship between socioeconomic background and mental health problems [1]: socioeconomically disadvantaged adolescents are two to three times more likely to develop mental health problems [2,3,4]. These inequalities are driven by complex and interrelated factors. Several studies over the years have indicated that the selection and causation effects are not mutually exclusive; rather these processes a cycle of deprivation and mental health problems that persists across generations [3,5]. Irrespective of socioeconomic status, adolescents should have access to and receive mental health services according to their needs. Studies, however, also reveal significant variations in the utilisation of mental health services among individuals, including adolescents, by their socioeconomic status (SES), the environments in which they live, and their capability to access available services [5,6,7,8,9].

Estimates from national surveys in Australia show that about 14% of adolescents suffer from mild to severe mental health disorders, the most common ones being attention-deficit-hyperactivity disorder and anxiety disorder [10,11]. Despite the availability of effective services delivered by the mixed public-private health system, a concerning proportion of adolescents in the country have unmet mental health needs and remain untreated [12] (Sheppard, Deane et al., 2018). Only 65% of adolescents aged 12–17 years old with a mental disorder in the past year sought care or spoke to a health professional about their symptoms [13]. Among the different kinds of available services, health professionals and online services were accessed more frequently, followed by school and telephone services [13,14].

The most common socio-demographic factors that influence mental health service utilisation among adolescents, identified across Australia and other developed nations, include gender [15,16,17], age [16,18], household/parental income [17], parental education and employment [14], location and ethnicity/immigration status [19,20]. Moreover, Vu, Biswas et al. [14] and Radez, Reardon et al. [21] revealed differences in access to services based on family types with children from blended, step-, and sole-parent households compared to original-parent households being more likely to use any type of mental health services. However, the majority of the previous research has been focused on overall service usage by adolescents and limited research investigates the impact of individual and family-related factors on each available service and the number of services accessed by adolescents aged 13–17 years.

Furthermore, regardless of the high number of adolescents with unmet needs in Australia, we found the body of research on socioeconomic inequality in mental health services’ use in adolescents, especially using advanced analytical approaches such as concentration indices, to be scarce. A recent study by Bartram and Stewart [22], using nationally representative data among adults in Australia, found the utilisation of psychologist services to be more concentrated at higher income levels (i.e., pro-rich) and the distribution of unmet needs for psychotherapy (as a negative indicator of access) to be more concentrated at lower income levels (i.e., pro-poor) despite expanded public insurance coverage.

In this paper, we used the corrected Erreygers’ concentration index approach to measure socioeconomic inequality in each mental health service and identified the determinants of four different mental health services and the number of mental health services accessed by adolescents aged 13–17 years. We believe that the exploration of service use in a more sophisticated way will provide a greater understanding of the relationship between SES and mental health service use in adolescents.

## 2. Subjects and Methodology

### 2.1. Data Source and Sample Size

This study is based on a de-identified secondary dataset available from the Young Minds Matter (YMM) nationwide survey conducted between May 2013 and April 2014, which supplies the most reliable and comprehensive source of data till date on mental health and well-being among Australian children and adolescents [23]. The YMM is cross-sectional in design and follows a multi-stage, area-based random sampling technique to represent a sample of households across the country [10,24]. In total, 6310 parents of children aged 4–17 years (55% of eligible households) willingly completed a structured computer-based survey questionnaire via face-to-face interview. In addition, a tab-based, self-reported questionnaire was completed privately at home for 2967 children aged 11–17 years (89% of eligible households) to gather information on the health risk behaviours in the past 12 months before the survey. All the study participants provided written informed consent before data collection. For households with more than one qualifying child, the sample included one child. The sample excluded the most remote areas, homeless adolescents and adolescents living in residential care and families that could not supply an interview in the English language. Out of the sampled adolescents, only those aged between 13 and 17 years were considered for this paper (*n* = 2268), as the self-reported child data on service use were strictly limited to 13–17-year-olds [10,24].

The YMM was conducted by the Telethon Kids Institute, the University of Western Australia in partnership with Roy Morgan Research, and the Australian Government Department of Health. Ethics was obtained through the Human Research Ethics Committees (HREC) of the University of Western Australia and the Australian Government Department of Health (RA/4/1/9197, Project 17/2012). In addition, the authorship team obtained ethics approval from the HREC of the University of Southern Queensland for further research using YMM data (HREC Approval No. H16REA205). More detail about the YMM study design and data collection procedure can be found elsewhere [24].

### 2.2. Outcome Variables

Mental health service accessed by adolescents aged 13–17 years was considered as the outcome variable. Both parent data and self-reported child data provided information on the utilisation of the following services: (i) health services—any mental health-related services provided by the general medical practitioners, family physicians, paediatricians, psychiatrists, psychologists, psychotherapists, mental health counsellors, nurses and social workers, mental health support centres such as headspace centres and community clinics; (ii) school services—counselling service provided to a child at any school or in an educational institute; (iii) telephone service—when a child receives psychological counselling support over the phone; (iv) online services [13]. In the analysis, both parent data and self-reported child data were combined to create a dichotomous variable for each service and responses were included ‘Yes’ (coded as 1) and ‘No’ (coded as 0). Moreover, a new categorical variable was created as ‘number of services accessed’ with children who did not access any services (coded as 0), accessed a single service (coded as 1), and accessed multiple (two or more) services (coded as 2).

### 2.3. Explanatory Variables

Sociodemographic covariates included the age of the child (continuous variable), gender (boys and girls), country of birth (overseas and Australia), place of residence (regional/remote and major cities), education of parents (Year 10/11, diploma and bachelor), employment of parents (unemployed and employed), family type (original parents and others included step, blended, sole or foster parents), family cohesion (good and poor). Family cohesion variable was measured by the item: ‘How the family members get along with each other?’, using a Likert scale of very good, good, fair, poor, and very poor. Response options, ‘very good’ or ‘good’ were categorised as ‘Good’ (coded as 1), and responses ‘fair’, ‘poor’ and ‘very poor’ were categorised as ‘Poor’ (coded as 0). The equivalised household income quintiles were calculated by using an equivalence factor based on the ‘Modified OECD’ equivalence scale [25]. The equivalised household income quintiles were Q1: poorest, Q2: 2nd poorest, Q3: middle, Q4: 2nd richest and Q5: richest.

### 2.4. Statistical Analysis

The sociodemographic characteristics of the sample (*n* = 2268) were described using frequencies and percentages. Random effect logistic models were used to examine the association between each sociodemographic characteristic and mental health services. Factors yielding a *p*-value of less than 0.05 in the unadjusted models were included in the adjusted models. To measure socioeconomic inequality in the use of mental health services, concentration indices (CIs) were computed for each outcome variable. The value of CI is a summary measure of socio-economic inequality that ranges between +1 and −1 (i.e., − 1 ≤ CI ≤ 1), where a value of 0 (zero) shows no inequality. A positive value of the CI suggests inequality concentrated among the richest while the negative value indicates the disproportionate concentration amongst the poorest. The larger the absolute value of the CI, the greater the extent of inequality [26,27,28].

However, in the case of binary outcomes (e.g., whether a child accessed mental health services or not), CI values differ with the upper and lowest limits [26], as their mean varies over time and populations, which can lead to unreliable comparisons of inequalities [29,30]. Typically, two potential approaches are used to deal with this kind of issue: (i) the Wagstaff approach—standardising CIs by dividing with one minus the means of mental health services variables [27], and (ii) the corrected Erreygers’ approach—adjusting CIs by multiplying it by four times with the means of mental health services variables [30]. In the present study, the latter approach was used which satisfied all four properties of the rank-dependent variable of inequalities [31]. All analyses were performed in Stata software version 14.1.

## 3. Results

Table 1 portrays sample characteristics. The mean age of the study sample was 15.4 (SD = 1.38), more than half were boys (51.9%), the majority of the sample was from Australia (85.1%) and almost two-thirds (64.7%) were living in major cities. A higher percentage of adolescents had educated parents (68.2%, diploma and above), and employed parents (76.3%). About 41% of adolescents belonged to a blended family type, and around 81% reported good family cohesion. Most of the adolescents were from middle- to high-income households (62%, combination of quintile 1, quintile 2 and quintile 3).

**Figure 1:**
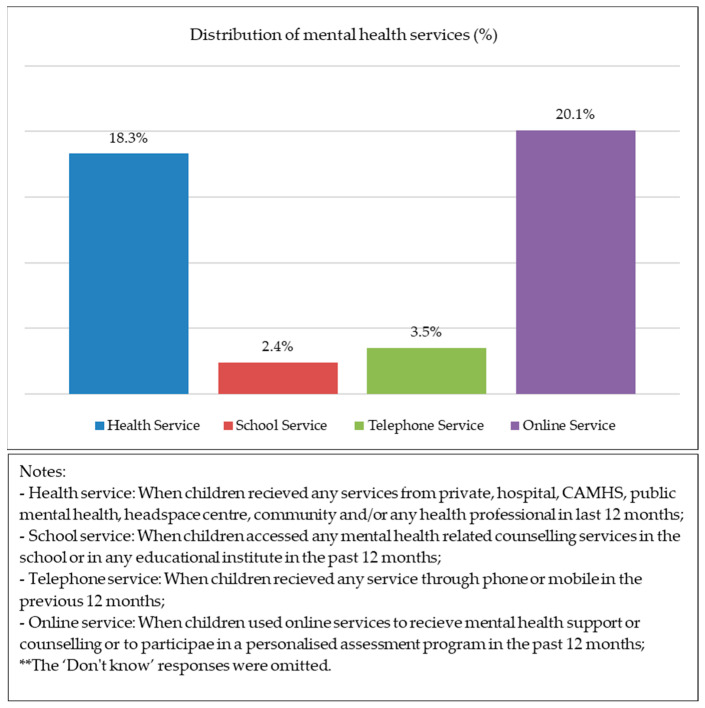
Distribution of four different mental health services.

**Figure 2:**
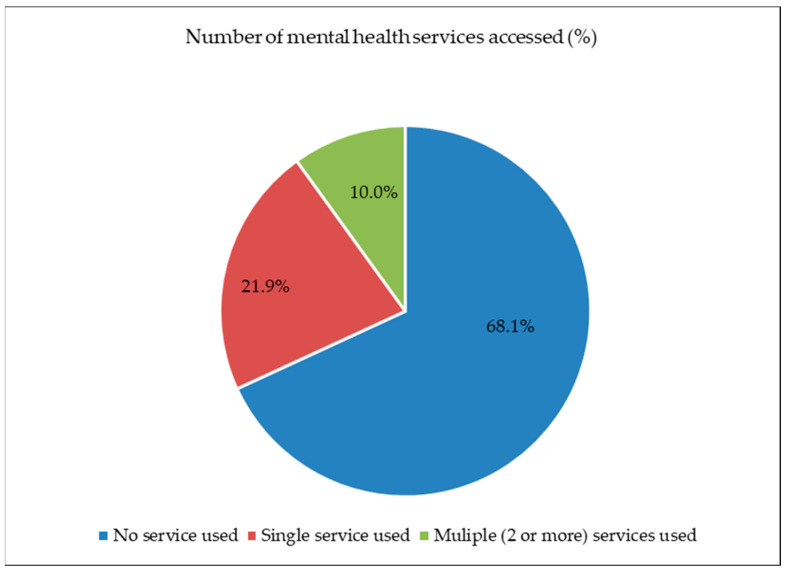
Percentages of the number of mental health services accessed.

Figure 1 illustrates the percentage distribution of the use of mental health services by Australian adolescents aged 13–17 years. Out of the four services, online services (20.1%) were preferable, and school services (2.4%) were the least popular service among adolescents, while approximately 18.3% of adolescents used general health services and 3.5% used telephone counselling services. Figure 2 shows that about nearly 22% of the sample accessed single services and 10.0% used two or more mental health services.

Figure 2 shows that about nearly 22% of the sample accessed single services and 10.0% used two or more mental health services.

Adjusted random effects model in Table 2 reveals children with increased age (aOR: 1.10, 95% CI 1.01–1.19), being girls (aOR: 1.67, 95% CI 1.34–2.08), being born in Australia (aOR: 1.65, 95% CI 1.16–2.35), living in a blended family (aOR: 1.72, 95% CI 1.36–2.18), and children with poor family cohesion (aOR: 1.31, 95% CI 0.99–1.71) were more likely to use general health services compared to their respective counterparts. Table 2 also shows that no factors were significantly associated with school services, while being girls (aOR: 2.29, 95% CI 1.41–3.73), and blended-family types (aOR: 2.36, 95% CI 1.46–3.78) were significantly associated with the higher use of telephone counselling services compared to boys and original-family type, respectively. The adjusted model for online services in Table 2 also demonstrates that children with increased age (aOR: 1.15, 95% CI 1.06–1.25), being girls (aOR: 2.38, 95% CI 1.90–2.99), living in major cities (aOR: 1.31, 95% CI 1.00–1.71), and children with educated parents (Diploma, aOR: 1.34, 95% CI 1.01–1.77; Bachelor, aOR: 1.37, 95% CI 1.02–1.84) were more likely to increase the probability of using online services than their respective counterparts.

Table 3 depicts the factors associated with single- and multiple-service usage by the sample population. Girls and children living in a blended family or with stepparents significantly increases the likelihood of using both single and multiple mental health services compared to boys and those who were living in the original family or with biological parents, respectively. In addition, the increased age of the study child and poor family cohesion were also found to be significantly associated with the higher use of multiple services compared to their counterparts.

The concentration indices (CIs) for four mental health services in Table 4 suggests that adolescents from economically worse-off households were more likely to use general health services (CI = −0.073, *p* < 0.001) and telephone counselling services (CI = −0.032, *p* < 0.001) than those who were economically better off.

## 4. Discussion

The Australian Government has made substantial investments in implementing extensive interventions aimed at delivering mental health services to mitigate mental health issues and their associated consequences among adolescents [32]. The impetus behind this initiative stemmed from the observation that a significant number of adolescents do not receive timely mental health care owing to a lack of services or access restrictions [11,33,34,35]. The current study sheds light on the key determinants of four different mental health services (general health service, school service, telephone service and online services), the factors associated with the number of mental health services accessed (single and multiple), and the socioeconomic inequalities in the usage of four different mental health services by Australian adolescents aged 13–17 years. Overall, this study revealed that older age groups, being a girl, living in major cities, being a child from a step/blended families or a child with poor family cohesion are factors that significantly increased use of mental health services compared to their counterparts, and socioeconomic inequalities exist in the use of general mental health services and telehealth services.

Despite previous research [36,37] showing a decline in overall mental health service use from mid-adolescence (14–15 years), this study found that the use of general mental health, online, and multiple mental health services increased with the higher age of study compared to younger ones. This is maybe because the older ones have internet access, and have a better idea about the service availability compared to the younger age group [14]. This study also indicates that the age of study was not significantly associated with school services and telephone services, maybe due to social stigma and embarrassment, as reported in previous studies [21].

Moreover, this study found that girls were more likely than boys to use mental health services (general health, telephone, online, single and/or multiple services) except school services. As reported in the published literature, this is maybe due to genetic and biological factors. For instance, research suggests that possibly due to hormonal fluctuations during the menstrual cycles, girls experience mood swings and eventually may develop anxiety/depression and seek mental health services [38,39,40].

Moreover, along with other studies [21,41,42,43], this study also revealed that children from step/blended families and children with poor family cohesion were more likely to use health, telephone and multiple mental health services compared to their counterparts. There is a possibility that adolescents belonging to these families may encounter an increased likelihood of mental health disorders, leading them to be more inclined towards seeking assistance compared to those residing with their biological families or families characterised by strong cohesion.

Our study also found that adolescents living in major cities used more online services than those who were living in regional/remote areas. This could primarily be because adolescents living in major cities had more access to modern hi-tech devices such as smartphones, iPad/tabs, and laptops with very good internet connections for their personal use than adolescents living in regional/remote areas [44,45,46,47].

Furthermore, the findings of the study revealed the existence of socioeconomic inequalities in general mental health and telehealth services usage within the same sample. In this study, we measured the equivalised household income-based socioeconomic inequalities by adopting the concentration index (CI) approach, which assisted us in gaining a deeper understanding of the underlying causes of socioeconomic inequalities in mental health services utilisation in our communities, which is essential from policy perspectives [48]. Evidence suggests that income-based health-related inequalities in Australia are both considerable and persistent [49]; as a result, the Government of Australia launched a country-wide programme (i.e., Better Access to Mental Health Care) in 2006 [22,50]. Consistent with previous studies from other developed countries including the USA, the UK and Australia [48,51,52,53,54], the results indicate pro-rich (in health and telephone services) inequalities in the utilisation of mental health services in Australia; however, the extent of inequality found was small.

Although the current study did not estimate the prevalence of mental health problems in the sample by socioeconomic status, an earlier study reported that children and adolescents from poorer families usually live in harmful conditions of abuse, crime, social strife, civil unrest, homelessness, and unemployment which places them at a higher risk for psychological distress and mental illnesses. Moreover, research suggests poor neighbourhoods also seem to have much greater effects on mental illnesses than well-to-do families [49]. Another study conducted in Spain reported that under-15-year-old adolescents from lower socioeconomic status accessed more mental health services compared to children belonging to higher socioeconomic families [42], while a population-based cohort study in Denmark claimed that people from low-income backgrounds accessed fewer mental healthcare services compared to high-income ones [55]. A study from Australia, on the other hand, reported that respondents from low-income backgrounds were more likely to use health services (e.g., general practitioners), but were less likely to use other healthcare services for preventive purposes such as mental health counselling for self-harm/suicidality, pap-smear, mammography for breast cancer, etc. [50].

There are several policy implications of this study, which can be contextualised nationally and globally. For example, public health researchers and policymakers should address inequality in mental health services’ use among children and adolescents as it is a matter of concern that, although Better Access was launched in 2006, inequality persists in Australia. In addition, Government policy should be structured in such a way that children and adolescents can obtain adequate psychological counselling support online, particularly focusing on those from lower socioeconomic backgrounds. Moreover, since this study has found some socioeconomic factors that affect mental health service utilisation among adolescents, it would be worthwhile to conduct an inequality analysis to track down the progress toward equality in service use.

Although this study has used the latest child and adolescent mental health survey data in Australia, this study also has some limitations. First, the main outcome of this study, access to mental health services, is likely to be subject to recall bias and social desirability bias as the YMM study used self-reported child- and parent-reported information. Additionally, causal interpretations were not possible due to the cross-sectional study design. Further, as this study only covers adolescents aged 13–17 years, the study findings may not be generalisable for other age groups such as children aged less than 13 years, young adults, and adults, who make up a significant proportion of the Australian population. Moreover, this study lacks information on the distribution of mental health service use among the indigenous and refugee populations, which might have additional policy implications. Furthermore, since the data were collected using a computer-based questionnaire, access to technology and self-completion of the questionnaire might be an issue for participants from disadvantaged groups, which may under/overestimate the findings.

## 5. Conclusions

The study revealed age, gender, family type and family cohesion were the key determinants of general mental health-, online-, and multiple mental health-service usage. Further, when compared to adolescents from higher socioeconomic backgrounds, those from lower socioeconomic backgrounds were more likely to make use of general mental health services and telephone services in Australia, implying pro-rich inequalities even though the magnitude of inequality was small. The study also found that targeting interventions specifically for low-income adolescent populations can be the most effective way to improve their use of mental health care services. In addition, from a policy perspective, a tailored as well as holistic approach is required to widen the knowledge about the determinants and inequalities of mental health services.

These findings will help in guiding mental health planners and policymakers in developing effective mental health services that can be accessed and used by all those who need them the most.

## Figures and Tables

**Table 1:** Sample characteristics.

Characteristics	*n*	%
Age	Mean = 15.42, SD = 1.38	
Gender		
Boys	1177	51.9
Girls	1091	48.1
Country of Birth		
Overseas	339	14.9
Australia	1929	85.1
Place of residence		
Regional/remote	801	35.3
Major cities	1467	64.7
Parental education		
Year 10/11	722	31.8
Diploma	819	36.1
Bachelor	727	32.1
Parental employment		
Employed	1730	76.3
Unemployed	538	23.7
Family type		
Original	1339	59.0
Blended and others	929	41.0
Family cohesion		
Good	1853	81.7
Poor	415	18.3
Household income quintile		
Q1 (0–20%)	402	17.2
Q2 (20–40%)	473	20.9
Q3 (40–60%)	400	17.6
Q4 (60–80%)	543	23.9
Q5 (80–100%)	450	19.8

**Table 2:** Determinants of each mental health services (*n* = 2268).

	Health Service		School Service		Telephone Service		Online Service	
	Unadjusted OR (95% CI)	Adjusted OR (95% OR)	Unadjusted OR (95% CI)	Adjusted OR (95% OR)	Unadjusted OR (95% CI)	Adjusted OR (95% OR)	Unadjusted OR (95% CI)	Adjusted OR (95% OR)
Age	1.11 ** (1.02–1.20)	1.10 * (1.01–1.19)	0.78 * (0.64–0.94)	-	1.02 (0.86–1.21)	-	1.15 *** (1.06–1.25)	1.15 ** (1.06–1.25)
Gender								
Boys	Ref	Ref	Ref	-	Ref	Ref	Ref	Ref
Girls	1.68 *** (1.35–2.09)	1.67 *** (1.34–2.08)	1.51 (0.87–2.62)		2.34 ** (1.44–3.80)	2.29 ** (1.41–3.73)	2.41 *** (1.93–3.02)	2.38 *** (1.90–2.99)
Country of birth								
Overseas	Ref	Ref	Ref	-	Ref	-	Ref	-
Australia	1.71 ** (1.20–2.41)	1.65 ** (1.16–2.35)	0.91 (0.43–1.91)		1.40 (0.68–2.87)		0.89 (0.67–1.21)	
Place of residence								
Regional/remote	Ref	-	Ref	-	Ref	-	Ref	Ref
Major cities	1.10 (0.86–1.39)		1.35 (0.72–2.52)		1.21 (0.72–1.99)		1.41 ** (1.09–1.83)	1.31 * (1.00–1.71)
Parental education						-		
Year 10/11	Ref	-	Ref	-	Ref		Ref	Ref
Diploma	1.02 (0.78–1.32)		1.03 (0.52–2.05)		0.94 (0.55–1.61)		1.34 (1.02–1.76)	1.34 (1.01–1.77)
Bachelor	0.93 (0.71–1.22)		1.22 (0.62–2.42)		0.77 (0.43–1.39)		1.40 (1.07–1.85)	1.37 (1.02–1.84)
Parental employment								
Unemployed	Ref	Ref	Ref	-	Ref	Ref	Ref	-
Employed	0.61 *** (0.48–0.78)	0.71 * (0.55–0.92)	0.88 (0.47–1.66)		0.53 * (0.33–0.86)	0.61 * (0.37–0.98)	1.07 (0.83–1.39)	
Family type								
Original	Ref	Ref	Ref	-	Ref	Ref	Ref	-
Blended and others	1.97 *** (1.58–2.44)	1.72 *** (1.36–2.18)	1.58 (0.91–2.75)		2.51 *** (1.57–4.02)	2.36 *** (1.46–3.78)	1.06 (0.85–1.32)	
Family cohesion								
Good	Ref	Ref	Ref	-	Ref	-	Ref	-
Poor	1.36 * (1.04–1.78)	1.31 * (0.99–1.71)	1.59 (0.84–2.99)		1.35 (0.78–2.34)		1.13 (0.85–1.49)	
Household income quintile				-		-		
Q1 (0–20%)	Ref	Ref	Ref		Ref		Ref	Ref
Q2 (20–40%)	0.79 (0.57–1.08)	1.01 (0.72–1.41)	0.93 (0.36–2.34)		0.84 (0.45–1.56)		1.62 (1.12–2.32)	1.59 (1.09–2.31)
Q3 (40–60%)	0.52 *** (0.36–0.75)	0.73 (0.49–1.09)	1.22 (0.49–3.03)		0.53 (0.25–1.10)		1.53 (1.05–2.24)	1.40 (0.95–2.06)
Q4 (60–80%)	0.53 *** (0.38–0.73)	0.80 (0.55–1.16)	1.07 (0.44–2.57)		0.52 (0.26–1.01)		1.16 (0.81–1.67)	1.08 (0.74–1.58)
Q5 (80–100%)	0.58 ** (0.41–0.82)	0.90 (0.61–1.32)	1.15 (0.47–2.84)		0.31 ** (0.13–0.71)		1.58 ** (1.09–2.29)	1.37 (0.93–2.03)

Notes: CI = confidence interval. Level of significance considered: *p* < 0.05 *, *p* < 0.01 **, *p* < 0.001 ***.

**Table 3:** Factors associated with the number of mental health service accessed.

	Single Service Accessed		Multiple (Two or More) Services Accessed	
	Unadjusted OR (95% CI)	Adjusted OR (95% OR)	Unadjusted OR (95% CI)	Adjusted OR (95% OR)
Age	1.04 (0.97–1.12)	-	1.15 * (1.03–1.28)	1.13 * (1.01–1.27)
Gender				
Boys	Ref	Ref	Ref	Ref
Girls	1.38 ** (1.13–1.68)	1.37 ** (1.12–1.68)	2.72 *** (2.00–3.69)	2.67 *** (1.95–3.63)
Country of birth				
Overseas	Ref	-	Ref	-
Australia	1.19 (0.89–1.59)		1.11 (0.73–1.68)	
Place of residence				
Regional/remote	Ref	-	Ref	Ref
Major cities	1.08 (0.87–1.33)		1.43 * (1.01–2.02)	1.36 (0.96–1.94)
Parental education				
Year 10/11	Ref	-	Ref	-
Diploma	1.02 (0.81–1.31)		1.21 (0.85–1.73)	
Bachelor	0.98 (0.76–1.26)		1.21 (0.84–1.75)	
Parental employment				
Unemployed	Ref	-	Ref	-
Employed	0.81 (0.64–1.02)		0.78 (0.56–1.08)	
Family type				
Original	Ref	Ref	Ref	Ref
Blended and others	1.27 * (1.04–1.55)	1.26 * (1.03–1.55)	1.63 ** (1.22–2.18)	1.59 ** (1.18–2.14)
Family cohesion				
Good	Ref	-	Ref	Ref
Poor	0.93 (0.71–1.21)		1.53 * (1.08–2.17)	1.51 * (1.06–2.16)
Household income quintile				
Q1 (0–20%)	Ref	-	Ref	-
Q2 (20–40%)	0.92 (0.67–1.26)		1.14 (0.73–1.78)	
Q3 (40–60%)	0.85 (0.61–1.18)		0.84 (0.51–1.37)	
Q4 (60–80%)	0.71 (0.51–0.96)		0.79 (0.50–1.26)	
Q5 (80–100%)	0.81 (0.59–1.12)		1.00 (0.62–1.59)	

Notes: CI = confidence interval. Level of significance considered: *p* < 0.05 *, *p* < 0.01 **, *p* < 0.001 ***.

**Table 4:** Inequalities in the utilisation of mental health services among Australian adolescents.

Services	Concentration Index (CI)	Standard Error (CI)	*p*-Value
By each service			
Health service	−0.073	0.018	<0.001
School service	0.005	0.008	0.474
Telephone service	−0.032	0.009	0.002
Online services	0.017	0.019	0.363

Notes: The corrected Erreygers’ concentration index (CI) was used.

## Data Availability

Young Minds Matter (YMM) survey datasets are available on request at the Australian Data Archive (ADA) repository. For detailed information about the application for the YMM data, please visit https://dataverse.ada.edu.au/.
